# A new CAD/CAM tooth mobility simulating model for dental in vitro investigations

**DOI:** 10.1007/s00784-023-05133-9

**Published:** 2023-07-06

**Authors:** Christoph J. Roser, Andreas Zenthöfer, Christopher J. Lux, Stefan Rues

**Affiliations:** 1 Department of Orthodontics and Dentofacial Orthopedics, Heidelberg University Hospital, Im Neuenheimer Feld 400, 69120 Heidelberg, Germany; 2 Department of Prosthodontics, Heidelberg University Hospital, University of Heidelberg, Im Neuenheimer Feld 400, 69120 Heidelberg, Germany

**Keywords:** Tooth mobility, Tooth model, CAD/CAM, Dental material, Chewing simulation

## Abstract

**Objectives:**

To validate a new tooth mobility simulating in vitro model for biomechanical tests of dental appliances and restorations.

**Material and methods:**

Load-deflection curves for teeth in CAD/CAM models (*n* = 10/group, 6 teeth/model) of the anterior segment of a lower jaw with either low tooth mobility (LM) or high tooth mobility (HM) were recorded with a universal testing device and a Periotest device. All teeth were tested before and after different ageing protocols. Finally, vertical load capacity (F_max_) was tested in all teeth.

**Results:**

At F = 100 N load, vertical/horizontal tooth deflections before ageing were 80 ± 10 µm/400 ± 40 µm for LM models and 130 ± 20 µm/610 ± 100 µm for HM models. Periotest values were 1.6 ± 1.4 for LM models and 5.5 ± 1.5 for HM models. These values were within the range of physiological tooth mobility. No visible damage occurred during ageing and simulated ageing had no significant effect on tooth mobility. F_max_ values were 494 ± 67 N (LM) and 388 ± 95 N (HM).

**Conclusion:**

The model is practical, easy to manufacture and can reliably simulate tooth mobility. The model was also validated for long-term testing, so is suitable for investigating various dental appliances and restorations such as retainers, brackets, dental bridges or trauma splints.

**Clinical relevance:**

Using this in-vitro model for high standardised investigations of various dental appliances and restorations can protect patients from unnecessary burdens in trials and practice.

## Introduction

In vitro testing is essential for establishing new materials and designs in dentistry. These tests are highly standardised and do not depend on patient’s compliance. They also allow designs and materials to be tested before clinical trials, which reduces costs and burden on patients. This is especially important in periodontological or trauma cases.

Models for biomechanical in vitro testing need to simulate the clinical situation as closely as possible. Simulating the mechanical behaviour of the periodontal ligament (PDL) is particularly challenging, but is mandatory since rigidly fixed teeth can bias the results in an in vitro model [1, 2]. Several efforts have been made to simulate natural tooth mobility in in vitro models, including techniques like socket enlargement, screw loosening, and simulation of alveolar bone loss [3]. Of these techniques, socket enlargement has been reported the most [1, 4–8]. In this model, space around the teeth is generated and subsequently filled with elastic materials that simulate the PDL [4–11]. However, the socket enlargement technique requires interradicular space, so cannot model anatomical areas like the mandibular anterior region, which has limited interradicular space. To overcome this problem, other models have reduced the artificial bone height to adjust tooth mobility [6, 9–14]. However, this has unpredictable and irreproducible outcomes [3]. This is also true for the screw loosening technique, which adjusts tooth mobility by loosening screws that hold the tooth in its socket [15, 16]. These current models are complex to manufacture and restricted to the simulation of anatomical areas with enough interradicular space. In addition, no model has shown reliable and unaltered simulation of tooth mobility after simulated ageing.

**Fig. 1:**
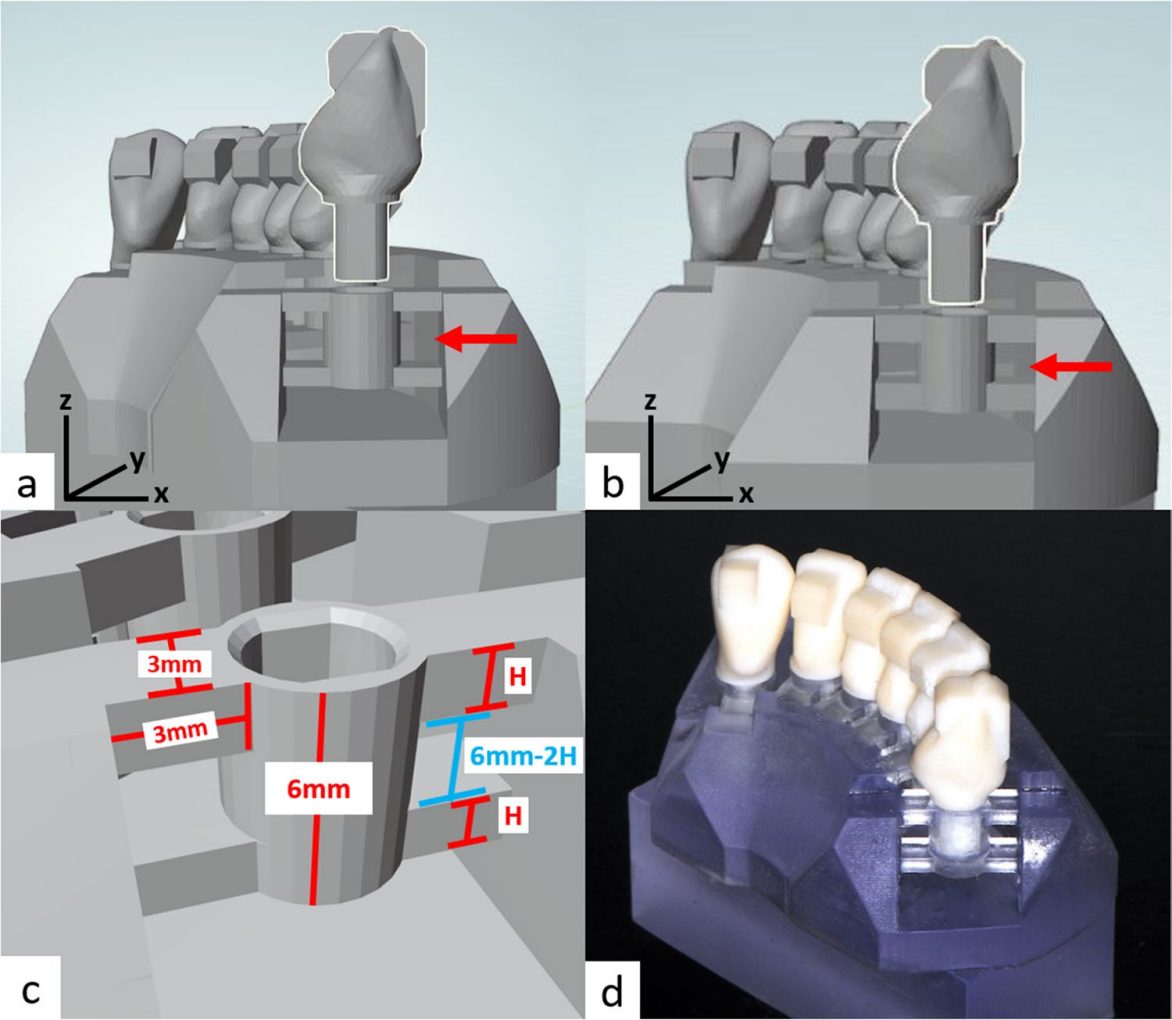
Model construction with respective dimensions. To simulate different tooth mobility values, two different models with different bar geometries and different inter-bar distances were designed (a = for high mobility; b = for low mobility). Design parameters are shown in c. To prevent rotation of the artificial teeth added to a model base, sockets and roots of the teeth were designed with each two flat surfaces on the mesial and distal side (**a**–**c**). All model bases were manufactured with a stereolithography printer and teeth milled from fibre-reinforced composite blanks. The artificial teeth were inserted and bonded in the respective tooth sockets (**d**)

The aim of the present study was to develop a novel dental in vitro model using CAD/CAM technology. For this purpose, a 3D printed model base with tooth sockets supported by bars was fitted with test teeth milled from fibre-reinforced composite (FRC). Tooth resilience was adapted by changing the bar heights (H, Fig. 1), which allowed the resilience of individual teeth to be easily adjusted in the model. We examined two bar heights leading to either a low physiological tooth mobility (LM, H = 1.7 mm) or a low tooth mobility (HM, H = 1.2 mm). Since in vitro models have to withstand artificial ageing procedures (water storage and chewing simulation), tooth resilience was recorded before and after artificial ageing and fracture resistance was determined at the end. The null hypotheses of this study were that tooth resilience would not be affected by model type (H1), tooth position (H2) and simulated ageing (H3).

## Methods

### Model construction and fabrication

A typodont model of a lower jaw (ANA 4, Frasaco, Tettnang, Germany) defined the crown geometries and spatial arrangement of the test teeth in the anterior segment of the lower jaw. A model base with the outline of an alveolar ridge and integrated support structures for the individual teeth was designed (Geomagic Design X; 3D Systems, Rock Hill, USA; Fig. 1b). Each 6 mm high support structure was connected to the alveolar ridge on the labial and oral side by two bars each and comprised a standardised socket for the “root” of the respective test tooth. Two different model bases, each with uniform support elements for the implemented test teeth, were used in this study. The first base had support elements that gave low tooth mobility (LM models, H = 1.7 mm), and the second had support elements that gave high tooth mobility (HM models, H = 1.2 mm). The gap between the lower side of the support structures and the base plate was 2 mm. A cement gap of 50 µm was implemented between socket and root. The vertical end position of the test tooth was defined by the horizontal plateau of the socket. Planar regions were added to the crown surfaces to standardize load application with the load vector tilted by either 0° (vertical), 45°, or 90° (horizontal) with respect to the tooth axis as well as predefined loading sites (uniform lever arms for each tooth with respect to the respective socket). Details are shown in Fig. 1b.

Test teeth (positions 3.3, 3.2, 3.1, 4.1, 4.2, and 4.3, *n* = 20 for each tooth type) were milled from FRC discs (Trinia, Bicon, Boston, USA). Teeth were nested such that the weak material was oriented in a mesio-distal direction. The ability of the FRC material to provide shear bond strength values (18.0 ± 2.4 MPa) in combination with adhesives used in dentistry comparable to the clinical situation was tested in advance according to DIN 13990 [17].

The model bases (*N* = 10/group) were fabricated from acrylic resin (Biomed Clear; Formlabs, Somerville, USA) in a stereolithography printer (Form 3B; Formlabs). Once printing was complete, cleaning, removal of support structures, and light curing were performed according to the manufacturer´s instructions.

Test teeth were jointed with the model bases by adhesive bonding. The socket and tooth root were both preconditioned by sandblasting (50 µm Al_2_O_3_, 1 bar) and by applying a primer (FRC: CRB Cera Resin Bond Set, Shofu, Ratingen Germany; acrylic resin: Visio Link, Bredent, Senden, Germany) before being connected by an adhesive (Tetric Evoflow, Ivoclar, Ellwangen, Germany).

### Testing of tooth mobility

**Fig. 2:**
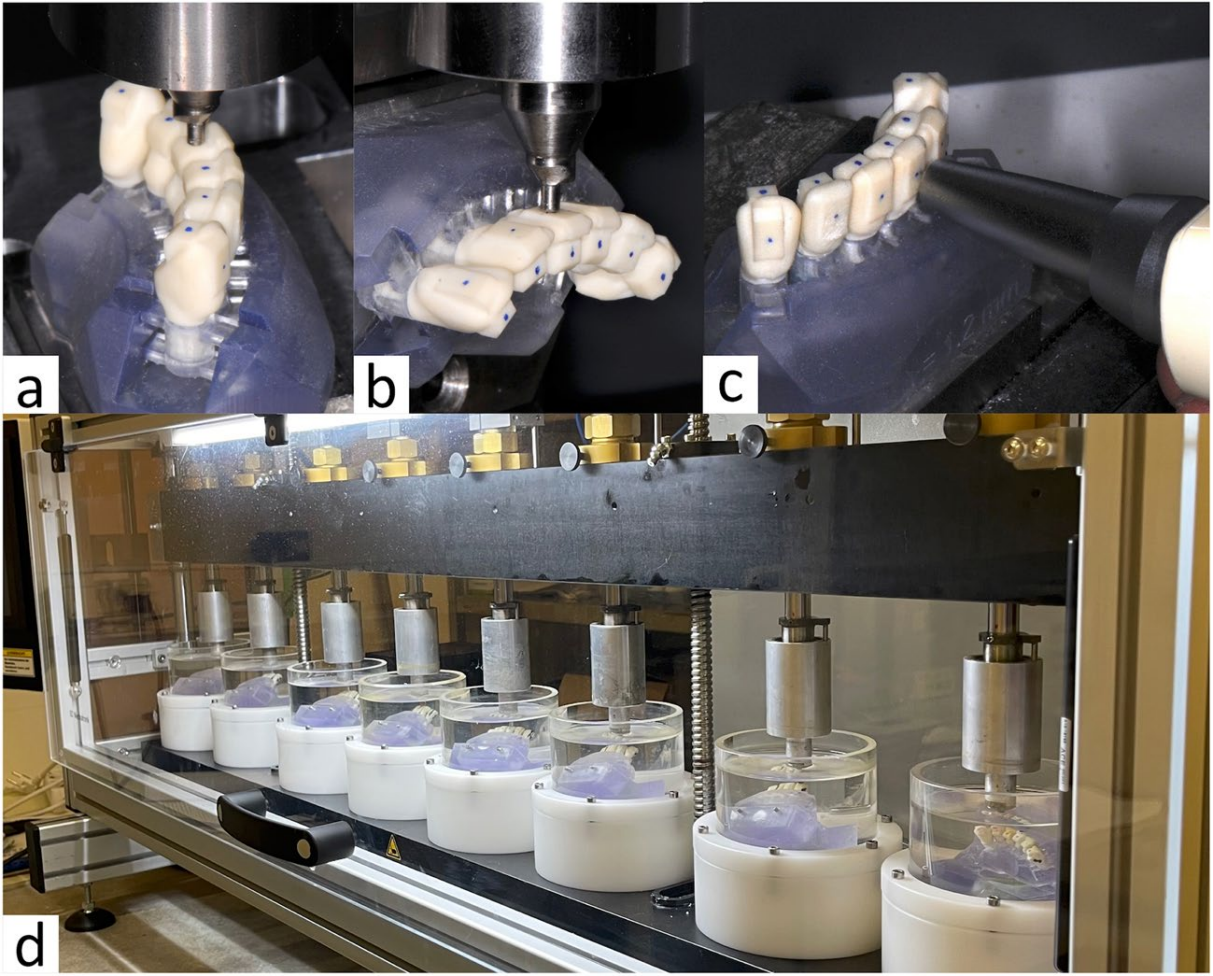
Tooth mobility testing (**a**-**c**) and ageing simulation (**d**). Vertical (**a**) and horizontal (**b**) tooth mobiltiy were tested with a universal testing device in all models at the exact centre of the respective loading surface. Moreover horizontal mobility measurements were performed with a Periotest device (**c**). After tooth mobility was tested the first time, all models were loaded 1,200,000 times with 64 N force magnitude in a chewing simulator (**d**) and stored afterwards in water at 37 °C until a total water storage time of 30 days was reached. Tooth mobility was then tested again followed by determination of the maximum loading capacity

Testing procedure is shown in Fig. 2a-c. Resilience of the models was tested by measuring displacements during vertical and horizontal loading in a universal testing device (Z005; Zwick Roell, ULM). Four loading cycles (three initial cycles followed by one measurement cycle) between 0 and 125 N (vertical loading) or 0 N and 70 N (horizontal loading) were conducted at a cross-head speed of 1 mm/min. To guarantee standardised and replicable load application, each load was applied at the exact centre of the respective loading surface, marked with a coloured dot in advance. Each load application point was located at the same distance from the model´s base.

**Fig. 3:**
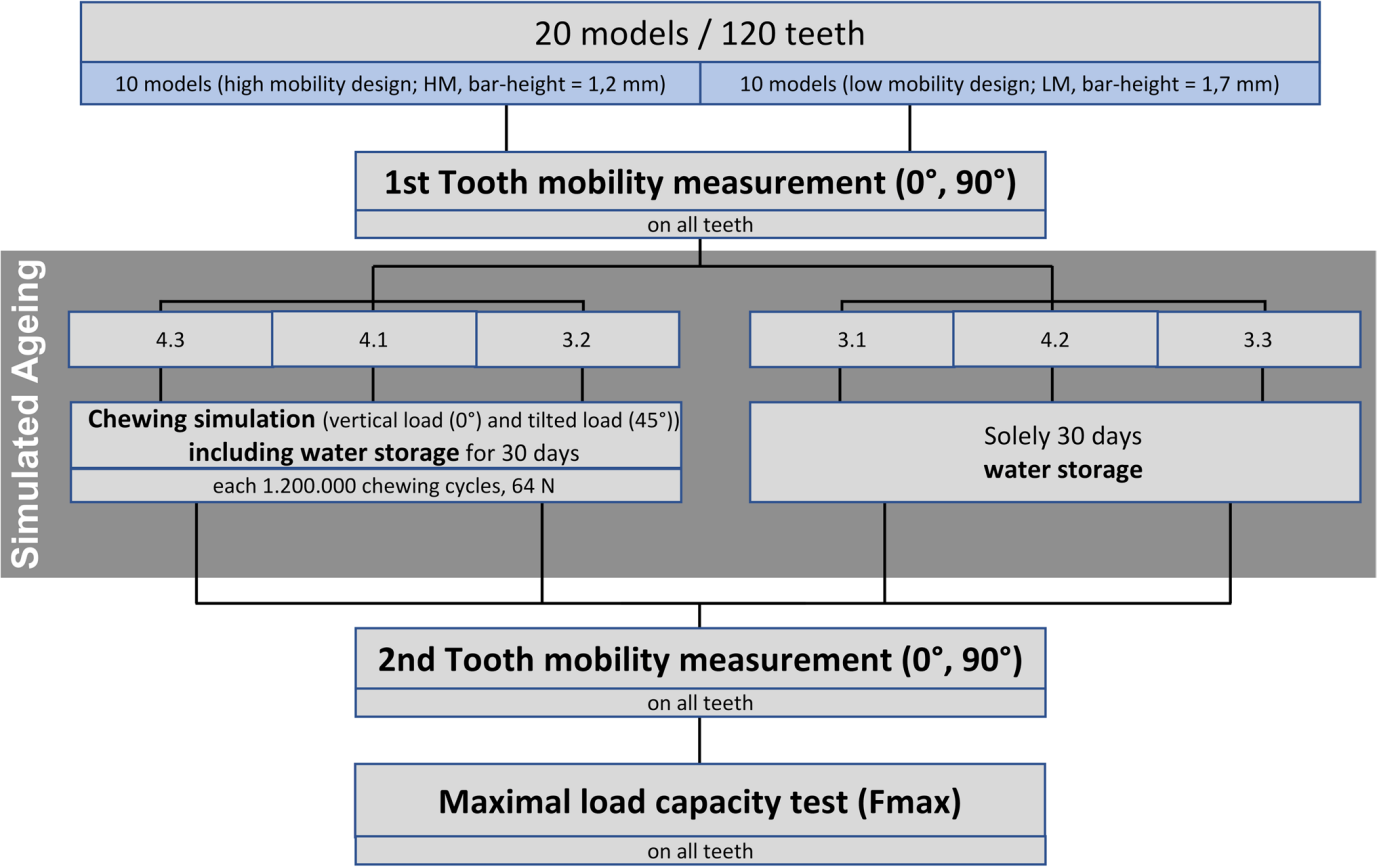
Study procedure. In total, vertical (0°) and horizontal (90°) tooth mobility were tested in 20 models/120 teeth before and after the simulated ageing process, which consisted of chewing simulation with samples immersed in water. For the chewing simulation, models were split into four groups according to their design (LM or HM) and according to the direction of load application (vertical, 0° or tilted, 45°). In the chewing simulator, models were successively loaded 1,200,000 times on each three of the six teeth with each simulation taking 10 days time. All models were loaded on teeth 4.3, 4.1, and 3.2. Finally, a maximum load capacity (F_max_) test was performed on all teeth. LM = low mobility; HM = high mobility

Deflections correlating with the actual test forces were measured and corrected by load deflection curves recorded for rigid samples with the test setup for horizontal as well as vertical loading. This compensated for deflections originating from the resilience of the test setup and the resilience of the test teeth could be calculated. This was done by fitting a line (method of least squares) to the loading aspect of the measurement cycle between 40 and 100 N for vertical loading or 20 N and 60 N for horizontal loading. The slope of this fitted line was the stiffness (k) for the respective tooth and the used loading condition. The correlating resilience was the reciprocal value (1/k). In addition, all models were horizontally tested on the same loading points with a Periotest device (Medizintechnik Gulden, Modautal, Germany) according to the manufacturer´s instructions (Fig. 2c). The Periotest device can measure tooth mobility quickly and easily in a clinical setting and works by applying percussive force to the tooth via a tapping device, which is then electromagnetically pulled back into the handpiece. Tooth mobility is quantified by the contact time between the tapping head and tooth [18, 19]. Tooth mobility was measured before and after a simulated ageing process. A flowchart of the complete testing procedure is shown in Fig. 3.

### Simulated ageing

After tooth mobility was measured for the first time before ageing (T0), all models underwent a standardised ageing process, which included 30 days of water storage at room temperature. During these 30 days of water storage, teeth 3.2, 4.1, and 4.3 were loaded sequentially with 1,200,000 chewing cycles and a force magnitude of 64 N (Fig. 2d; CS-4; SD Mechatronik, Feldkirchen-Westerham, Germany). Respective teeth of half of the models of each test group were loaded in vertical direction (0°), whereas respective teeth of the other half were loaded with the force vector tilted (45° with respect to the tooth axes) in labial direction. Thus, the artificial teeth could be split in three subgroups after simulated ageing (T1):

- T1_WS_ (*n* = 10/group, teeth 3.3, 3.1, 4.2): 30 days of water storage
- T1_0°CS_ (*n* = 5/group, teeth 3.2, 4.1, 4.3): 30 days water storage and axial (0°) chewing simulation
- T1_45°CS_ (*n* = 5/group, teeth 3.2, 4.1, 4.3): 30 days water storage and extra-axial (45°) chewing simulation.

### Further tooth mobility testing and maximum load capacity testing

To check whether any model components were damaged or had failed due to ageing, the models were visually inspected after the simulated ageing process. Then, further tooth mobility tests were performed as described above.

Finally, the vertical maximum load capacity (F_max_) was tested in all models with the universal testing device. Loads were applied on each tooth separately via a steel piston at a crosshead speed of 2 mm/min. Failure of the respective tooth/support structure assembly was defined by a drop-in test force of ≥ 20% F_max_.

### Statistical analysis

Statistical analysis was performed using SPSS 28 (IBM; Endicott, NY, USA). The null hypotheses on tooth resilience regarding model type and tooth position (H1 and H2) were analysed using two-way ANOVAs and Tukey post-hoc tests. Since subgroups for aged samples (T1) and tooth position would lead to small test group sizes, analysis of the factor tooth position was restricted to T0. The effect of simulated ageing (H3) was checked using paired-samples t-tests and Bonferroni corrections for multiple testing. The significance level was set to α = 0.05.

## Results

**Fig. 4:**
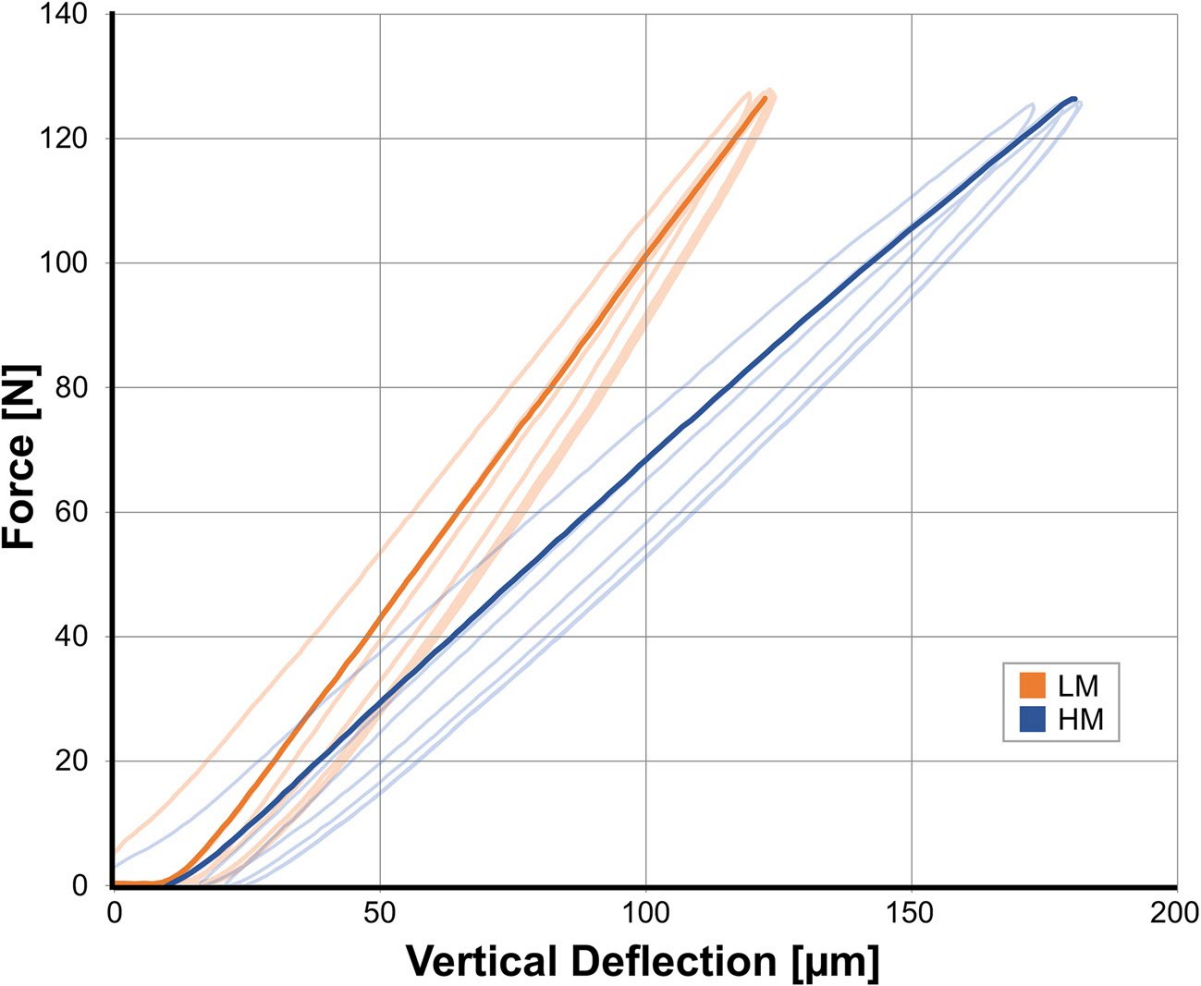
Representative load-deflection diagram for measuring vertical tooth mobility. In order to guarantee standardised and reliable measurement, three initial loading cycles were conducted before the main measurement

**Fig. 5:**
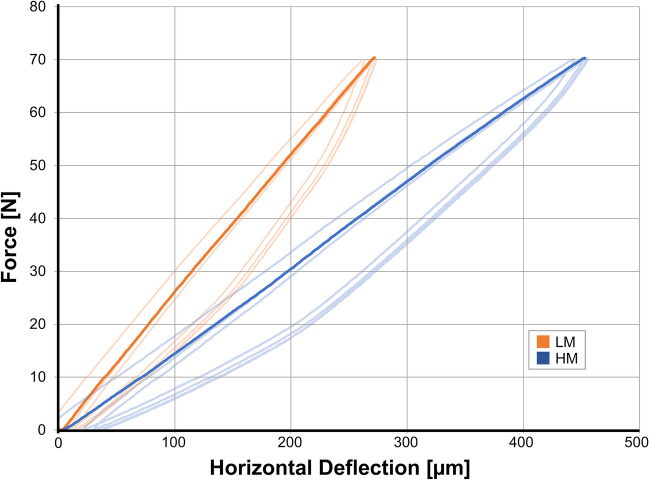
Representative load-deflection diagram for measuring horizontal tooth mobility. In order to guarantee standardised and reliable measurement, three initial cycles were conducted before the main measurement

Figures 4 and 5 show representative load deflection curves for both model groups. LM models had a mean vertical resilience of 83 ± 11 µm/100 N before simulated ageing and 82 ± 12 µm/100 N after simulated ageing. With respect to horizontal resilience, LM models showed a mean resilience of 400 ± 61 µm/100 N before and 386 ± 64 µm/100 N after simulated ageing. Corresponding mean Periotest values were 1.6 ± 1.4 for T0 and 1.5 ± 1.6 for T1 (Tables 1 and 2).

**Table 1:** Tooth mobility of low mobility (1.7 mm) models

Model	Tooth	Tooth resilience [µm/100 N]				Periotest values	
		*Vertical*		*Horizontal*			
		*T0*	*T1*	*T0*	*T1*	*T0*	*T1*
1.7 mm (low mobility)	*3.3*	85 ± 13	76 ± 8	407 ± 49	420 ± 57	2.1 ± 1.5	1.8 ± 1.6
	*3.2*	88 ± 10	85 ± 18	406 ± 52	383 ± 60	1.0 ± 1.1	1.1 ± 1.2
	*3.1*	85 ± 16	81 ± 13	392 ± 83	409 ± 95	1.6 ± 1.7	1.4 ± 1.6
	*4.1*	82 ± 9	85 ± 13	389 ± 60	366 ± 61	1.2 ± 1.0	1.4 ± 1.9
	*4.2*	81 ± 7	84 ± 9	372 ± 75	356 ± 49	1.4 ± 1.5	1.2 ± 1.1
	*4.3*	80 ± 11	85 ± 8	413 ± 42	384 ± 39	2.4 ± 1.0	2.5 ± 1.6
	*3.3–4.3*	83 ± 11	82 ± 12	369 ± 61	386 ± 64	1.6 ± 1.4	1.5 ± 1.6

**Table 2:** Tooth mobility of high mobility (1.2 mm) models

Model	Tooth	Tooth resilience [µm/100 N]				Periotest values	
		*Vertical*		*Horizontal*			
		*T0*	*T1*	*T0*	*T1*	*T0*	*T1*
1.2 mm (high mobility)	*3.3*	129 ± 25	119 ± 13	687 ± 119	635 ± 138	6.5 ± 1.6	6.3 ± 1.9
	*3.2*	129 ± 23	138 ± 34	576 ± 94	637 ± 169	5.7 ± 1.5	6.1 ± 1.3
	*3.1*	122 ± 18	149 ± 35	578 ± 70	526 ± 116	4.9 ± 1.4	5.1 ± 1.5
	*4.1*	126 ± 28	132 ± 22	626 ± 120	631 ± 168	5.0 ± 1.2	5.9 ± 1.9
	*4.2*	130 ± 22	143 ± 22	580 ± 90	585 ± 101	5.6 ± 1.5	5.0 ± 1.8
	*4.3*	124 ± 15	133 ± 32	614 ± 100	658 ± 124	5.9 ± 1.3	6.5 ± 2.0
	*3.3–4.3*	127 ± 21	136 ± 30	610 ± 100	611 ± 139	5.5 ± 1.5	5.7 ± 1.8

**Fig. 6:**
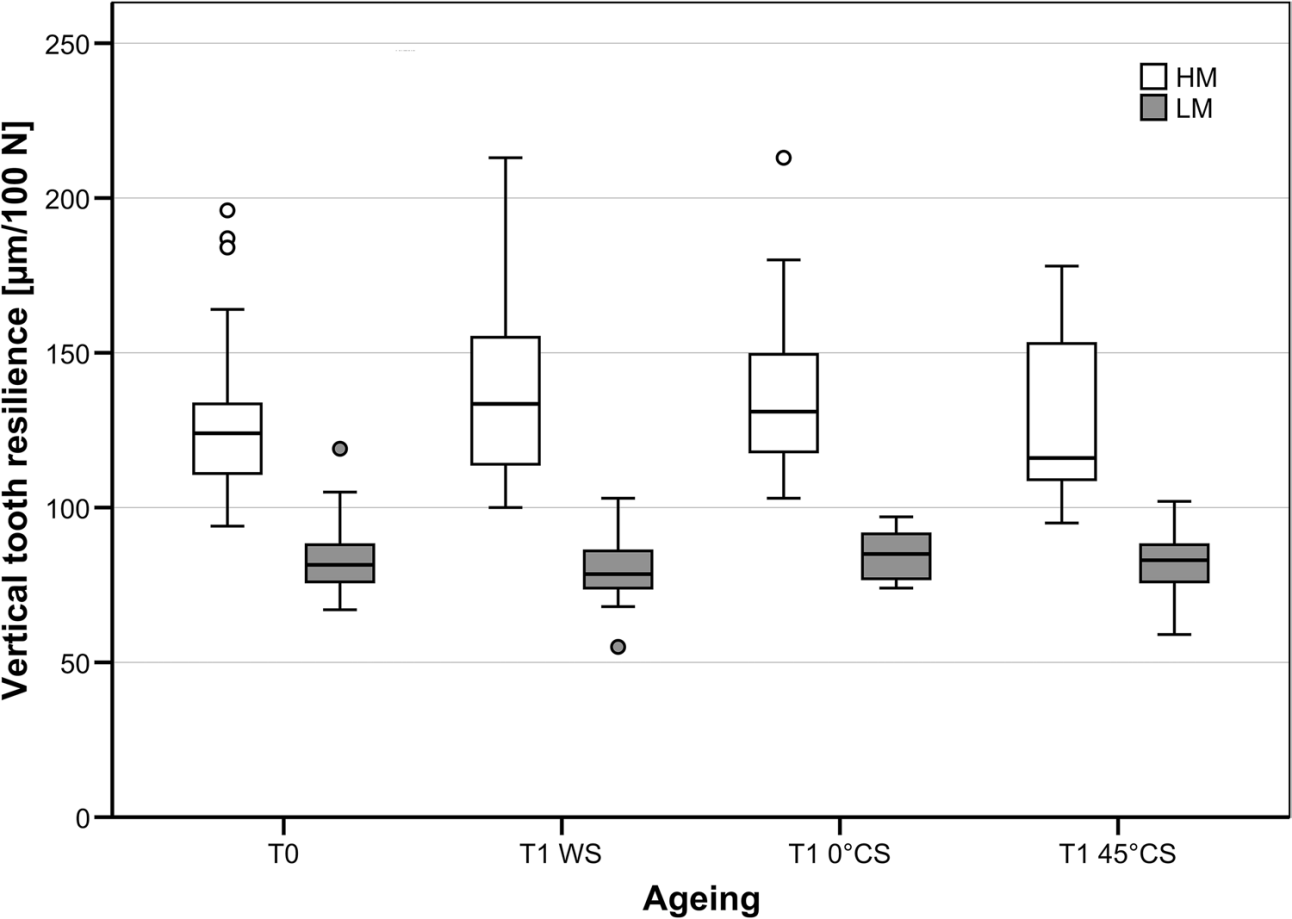
Effect of artificial ageing protocols on vertical tooth mobility. All models (HM: high mobility; LM: low mobility) showed reliable vertical tooth resilience during artificial ageing. None of the different ageing protocols had an influence on vertical tooth resilience. T1 WS, water storage; T1 0° CS, vertical chewing simulation in water; T1 45° CS, chewing simulation under 45° in water

**Fig. 7:**
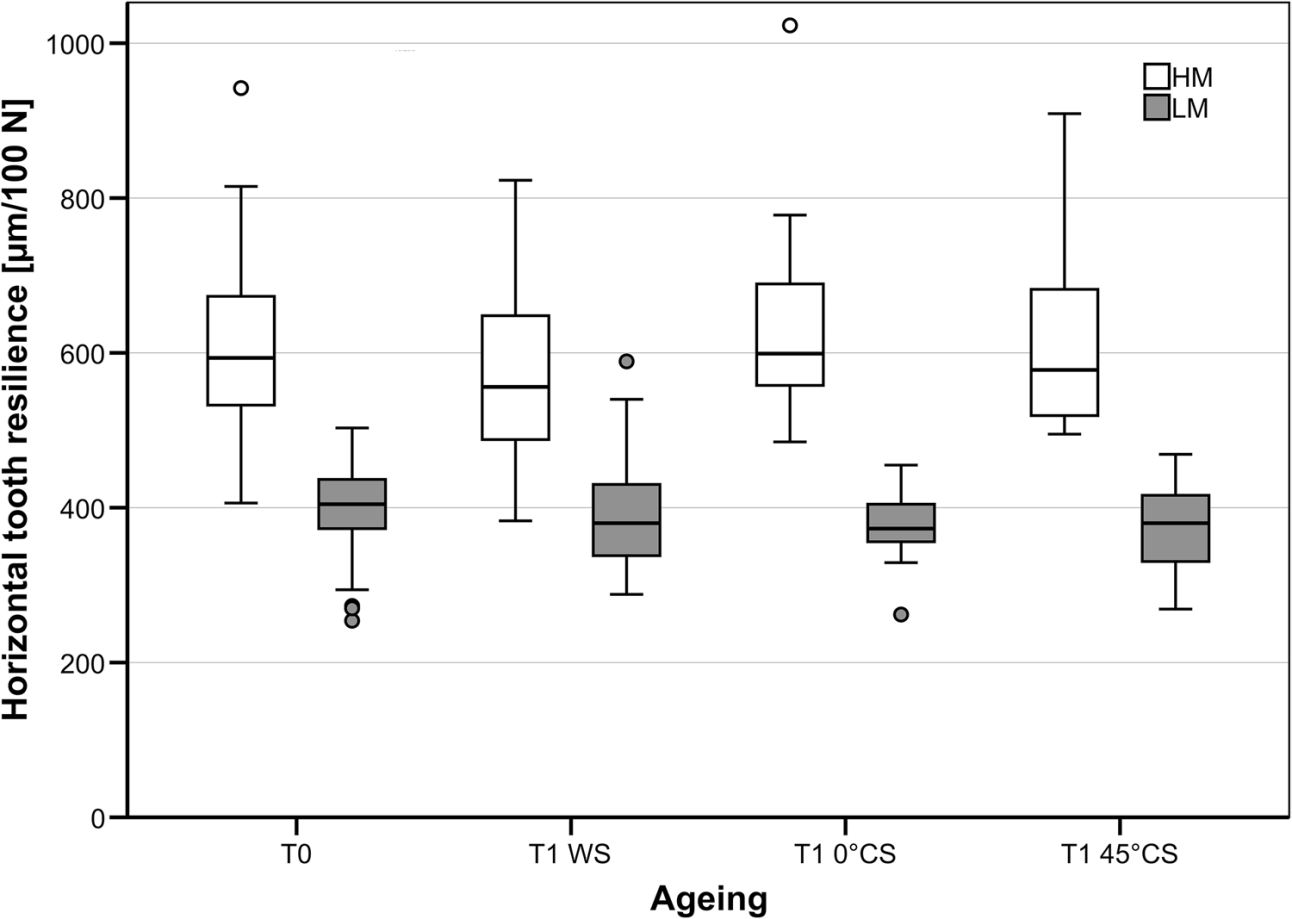
Effect of artificial ageing protocols on horizontal tooth mobility. All models (HM: high mobility; LM: low mobility) showed reliable horizontal tooth mobility during artificial ageing. Like for vertical tooth resilience, none of the different ageing protocols had an influence on horizontal tooth resilience. T1 WS, water storage; T1 0° CS, vertical chewing simulation in water; T1 45° CS, chewing simulation under 45° in water

No significant differences were found in vertical and horizontal resilience between the different teeth (*p* = *0.999*). Likewise, no significant differences were found in mean vertical and horizontal tooth resilience before and after simulated ageing (*p* = *0.055*). When comparing the different ageing protocols, none of the ageing protocols significantly influenced the vertical/horizontal tooth resilience of LM-models (T1_WS_: *p* = *0.801/p* = *0.230;* T1_0°CS_: *p* = *0.521/p* = *0.190*; T1_45°CS_: *p* = *0.045/0.941)* (Figs. 6 and 7)*.*

HM models had a mean vertical resilience of 127 ± 21 µm/100 N before simulated ageing and 136 ± 30 µm/100 N after simulated ageing. With respect to horizontal resilience, HM models showed mean values of 610 ± 100 µm before and 611 ± 139 µm after simulated ageing. Corresponding mean Periotest values were 5.5 ± 1.5 for T0 and 5.7 ± 1.8 for T1 (Tables 1 and 2).

No significant differences were found in vertical and horizontal tooth resilience between the different teeth (*p* = *0.982*). Likewise, no significant differences were found in mean vertical and horizontal resilience before and after the simulated ageing process (*p* = *0.464*) (Table 2). None of the ageing protocols had a significant effect on vertical/horizontal tooth resilience (T1_WS_: *p* = *0.170/p* = *0.106;* T1_0°CS_*: p* = *0.556/p* = *0.038*; T1_45°CS_: *p* = *0.510/p* = *0.465)* (Figs. 6 and 7)*.*

**Fig. 8:**
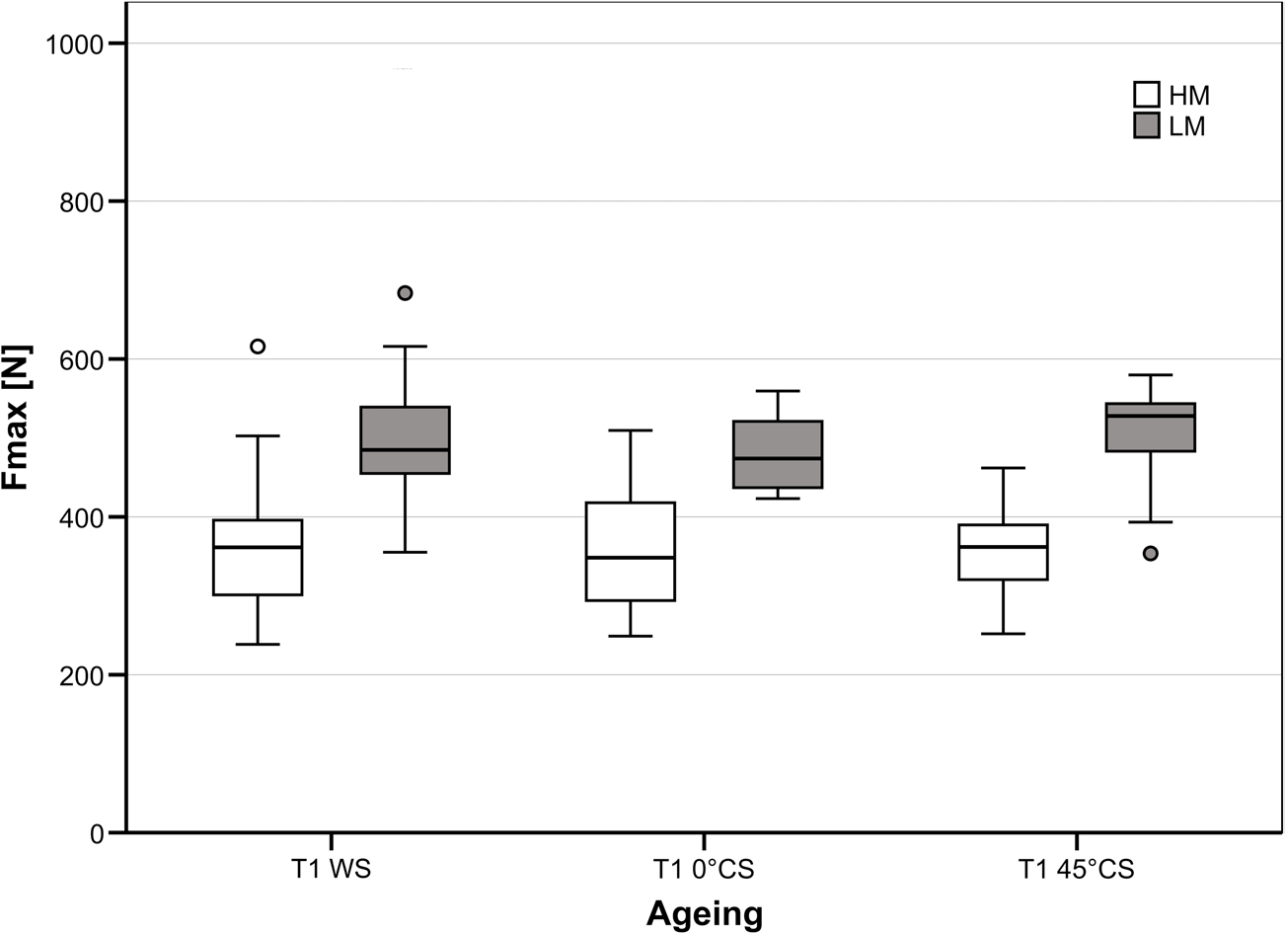
Maximum load capacity (F_max_) testing. High mobility (HM) models showed mean F_max_ values of 388 ± 95 N. Low mobility (LM) models had significantly higher F_max_ values (494 ± 67 N). The ageing protocols had no effect on F_max_ in the two models. T1 WS, water storage; T1 0° CS, vertical chewing simulation in water; T1 45° CS, chewing simulation under 45° in water

No model showed damage after the ageing process. With regard to F_max_, LM models had significantly higher (*p* < *0.001*) values (494 ± 67 N) than HM models (388 ± 95 N) (Fig. 8).

## Discussion

In this study, the first null hypothesis (H1) had to be rejected because tooth resilience was significantly different between HM and LM models. However, the other null hypotheses were confirmed because tooth resilience was not affected by tooth position (H2) or by simulated ageing (H3). These findings show that this model can reliably simulate physiological tooth resilience. Moreover, this model is suitable for long-term testing because simulated ageing did not affect tooth mobility or cause the model to fail.

As a methodical strength, in contrast to most of previous studies [4, 7, 9, 10, 14], we measured tooth mobility not only with a Periotest device, which is sensitive to factors like position and angulation [20], but also with a universal testing machine, which has only been done in a few other studies [11, 14]. Periotest measurements however were performed in the present study in order to provide the reader with a maximum of information, to allow comparability to other studies and to reference the clinical context. The Periotest values of our HM models were in the mid-range and the values of our LM models were slightly below the range of those measured in physiological teeth in vivo [18, 19]. This was also true for the metric resilience results of our universal testing device, which were in line with physiological tooth mobility values reported in another in vivo study [21]. However, an advantage of our CAD/CAM model is that it easily allows the setting of very individual mobility values, for example in patients with pre-existing conditions such as periodontitis or patient with a tooth trauma, simply by modifying the bar dimensions at the design level.

When comparing the presented model to previous models, it is noticeable that it is distinguished by its design and the related concept of tooth mobility simulation. Tooth mobility was previously adjusted by generating space circumferential to the root, which was then filled with elastic materials such as A-polyvinylsiloxane [8, 9], polyether [4], silicone [5, 10, 11] or latex-based rubber [6] to simulate the PDL. This space is usually created by applying a wax layer to the roots before the corresponding socket is produced. Tooth mobility can then be adjusted by changing the wax thickness [5, 8, 14]. However, this technique seems time consuming and is shown to be inconsistent in PDL thicknesses, ranging from 0.00 mm to 0.42 mm [6]. This seems high considering that teeth moved up to 0.69 mm in the present study. Several studies have tried to overcome this problem by applying artificial PDL material directly to the teeth [6]. Although this gives a more accurate thickness, it is still a time-consuming technique with limited replicability. Another drawback of previous studies is that they have not investigated whether their models can reliably measure tooth mobility after simulated ageing.

We have addressed these problems by introducing a simple, practical, and reliable method for adjusting tooth mobility. This was done by modifying the bars that hold the teeth within the model base. Because the design is fully digital, parameters can be accurately planned before in vitro testing. Moreover, intraoral scans and the associated stereolithography files allow patient-specific models to be produced in vitro, with individual tooth forms and individual tooth positions. This is useful in cases when design parameters need to be tested before a restauration or appliance is inserted. Another advantage of the model is that it is planned digitally, so in vitro tests can be combined with finite element analysis to plan test parameters precisely before in vitro testing is performed. The design is also highly standardised, so all adjustments can be made in one design and printed multiple times. This is in contrast to previous models, in which all adjustments had to be made on each model, creating high levels of variation.

Moreover, it has to be considered, that most of previous techniques limit the production of full dental models similar to the clinical situation because of the space needed to generate the PDL. This is particularly true for models of the mandibular anterior region. However, changing tooth geometry to construct smaller roots combined with bigger crowns leads to an unphysiological crown to root ratio with unphysiological biomechanical behaviour. Our model avoids these problems because it needs little additional interradicular space, so can simulate every dental situation. This was moreover facilitated by using artificial teeth made from FRC. Using either bovine or human teeth instead seemed rather unpracticable, because they are usually obtained from different sources and therefor differ with regard to their geometries and chemical compositions. This is why we previously validated FRC teeth for in vitro tests, finding sufficient F_max_ and clinical comparable shear bond strength values with particular low standard abbreviation [17]. Therefore, using FRC teeth in our model standardised both the model base and the teeth. Due to the advantages of the present model, we were already able to test orthodontic retainers precisely for their long-term and maximum stability [22]. We will moreover continue using it for investigations on other dental appliances and restorations.

A limitation of the present concept is that, although both model designs could withstand F_max_ values above the maximum physiological mastication force of about 270 N [23–27], they still have to be modified to withstand maximum load tests of appliances or restorations requiring higher F_max_ values. Importantly, changing design parameters to achieve higher mobility might affect the corresponding F_max_ values. Further studies are needed to investigate additional designs of this concept.

## Conclusions

This study presents a new dental CAD/CAM in vitro model for biomechanical in vitro testing, which:

- is particular precise and reliable in the simulation of physiological tooth mobility.
- allows easy adjustment of different tooth mobility values within the digital design process.
- is validated for long term testing with unaltered tooth mobility after simulated ageing.
- enables digital storage and multiple reproductions of a single design due to CAD/CAM production.

Therefore, it is suitable for the investigation of various dental appliances and restorations like brackets, retainers, dental bridges or trauma splints.

## Data Availability

Data available on request. The data underlying this article will be shared on reasonable request to the corresponding author.
